# Transesophageal Echocardiography Was Useful in Determining the Treatment Plan for Moderate Aortic Stenosis-Like Findings Associated With High Left Ventricular Output Immediately Following Biological Valve Replacement: A Case Report

**DOI:** 10.7759/cureus.111049

**Published:** 2026-06-17

**Authors:** Keisuke Sumii, Takumi Yamamoto

**Affiliations:** 1 Anesthesiology, Saitama Sekishinkai Hospital, Sayama, JPN; 2 Cardiovascular Disease, Saitama Sekishinkai Hospital, Sayama, JPN

**Keywords:** aortic valve replacement (avr), aortic valve stenosis (as), hyperdynamic state, patient prosthetic mismatch (ppm), transesophageal echocardiography (tee)

## Abstract

An elevated transvalvular pressure gradient and accelerated blood flow immediately following aortic valve replacement (AVR) is occasionally observed. It requires careful differentiation between structural factors, such as prosthetic valve dysfunction or patient-prosthetic mismatch (PPM), and non-structural factors, including high left ventricular output.

A 73-year-old woman presenting with severe aortic regurgitation underwent AVR with a 25 mm stented bioprosthetic valve and coronary artery bypass grafting. After weaning from cardiopulmonary bypass (CPB) under catecholamine support, intraoperative transesophageal echocardiography (TEE) revealed findings corresponding to moderate aortic stenosis (AS), with a maximum blood flow velocity of 3.4 m/s and a mean pressure gradient of 23 mmHg. However, further TEE evaluation demonstrated a normal effective orifice area index (EOAI) of 1.24 cm²/m² and a short blood flow acceleration time (AT) of 42 ms. Consequently, prosthetic valve dysfunction and PPM were ruled out, and these findings were attributed to a hyperdynamic state with high left ventricular output. Reoperation was successfully avoided. A follow-up transthoracic echocardiogram (TTE) on postoperative day 4 confirmed normal valve mobility without any signs of stenosis, and the patient was discharged without complications.

TEE played a crucial role in accurately identifying a high left ventricular output state masquerading as moderate prosthetic valve stenosis immediately following surgery, thereby preventing unnecessary surgical reintervention.

## Introduction

In rare cases, an aortic valve pressure gradient may be observed immediately following structural factors, for example, aortic valve replacement (AVR), indicating patient-prosthetic mismatch (PPM) or prosthetic valve dysfunction. It is often necessary to differentiate these conditions from those caused by non-structural factors, for example, surgical stress, anemia, or high left ventricular flow associated with catecholamine administration [1,2]. We report a case in which the patient presented with findings suggestive of moderate aortic valve stenosis (AS) due to high left ventricular output immediately following surgery. Using transesophageal echocardiography (TEE), it is possible to confirm whether typical structural problems, such as paravalvular leakage or valve dysfunction, exist, rule out functional problems, such as left ventricular hypercontractility or PPM, and verify whether left ventricular wall motion abnormalities have occurred due to stenosis or occlusion at the coronary artery ostia caused by the prosthetic valve.

## Case presentation

This case involves a 73-year-old woman, 151 cm, 60 kg, with a body surface area (BSA) of 1.56 m². She had been diagnosed with aortic regurgitation (AR) at our hospital 18 years prior but had been under observation without symptoms. A transthoracic echocardiogram (TTE) performed two months prior revealed reduced ejection fraction, and surgery was performed at the patient’s request. Preoperative TTE showed a left ventricular ejection fraction (LVEF) of 46% and circumferential left ventricular wall motion impairment. The angle between the aortic valve and mitral valve was 128 degrees. Severe AR with an eccentric jet was observed, characterized by a 6.8 mm vena contracta from a point slightly to the right of the mid-aortic valve toward the anterior mitral valve wall, with a pressure half-time (PHT) of 302 ms. The patient underwent AVR, and on-pump coronary artery bypass grafting was performed. A 25 mm stent-supported bioprosthetic valve was implanted to replace the aortic valve with a 27.3 mm annulus, and a bypass graft was created using a saphenous vein graft from the ascending aorta to the posterior descending branch of the right coronary artery.

**Figure 1:**
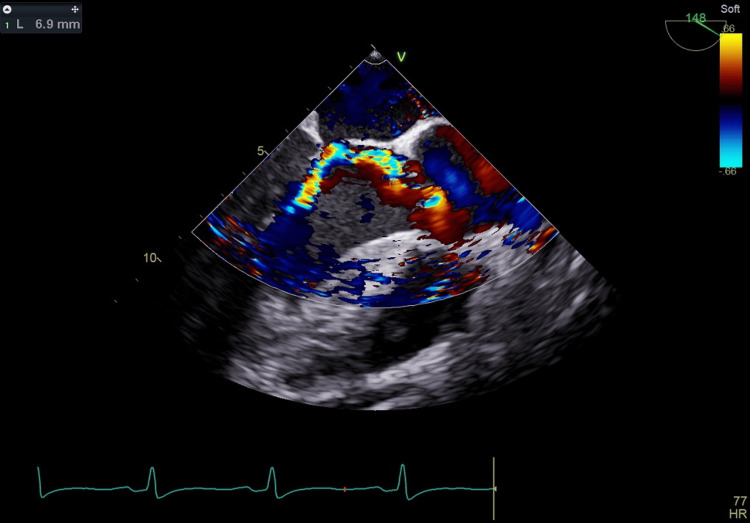
Severe AR assessed by the aortic valve long-axis view of TEE after induction of anesthesia A severe AR jet with an eccentric jet was observed from a point slightly to the right of the mid-aortic valve toward the anterior mitral valve wall. AR: aortic regurgitation; TEE: transesophageal echocardiography

**Figure 2:**
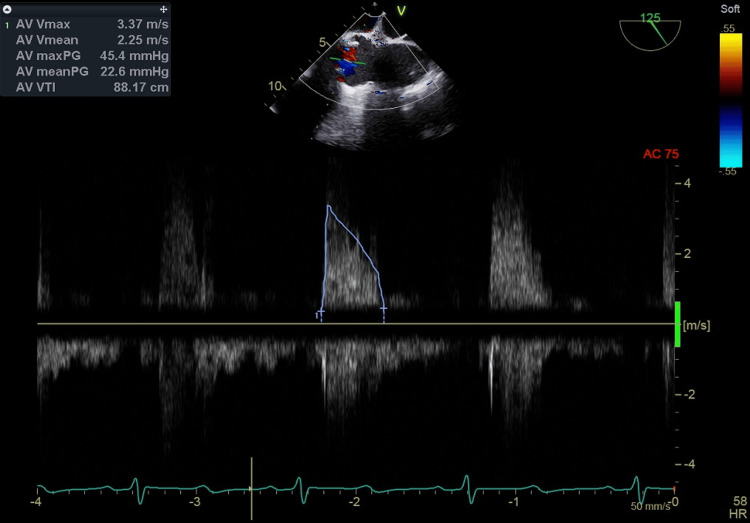
Assessment of blood flow velocity in accelerated blood flow corresponding to moderate AS by continuous wave Doppler of TEE after CPB It revealed a maximum aortic valve blood flow velocity of 3.4 m/s, a maximum aortic valve pressure gradient of 45 mmHg, and a mean aortic valve pressure gradient of 23 mmHg, indicating findings corresponding to moderate AS. AS: aortic stenosis; TEE: transesophageal echocardiography; CPB: cardiopulmonary bypass

**Figure 3:**
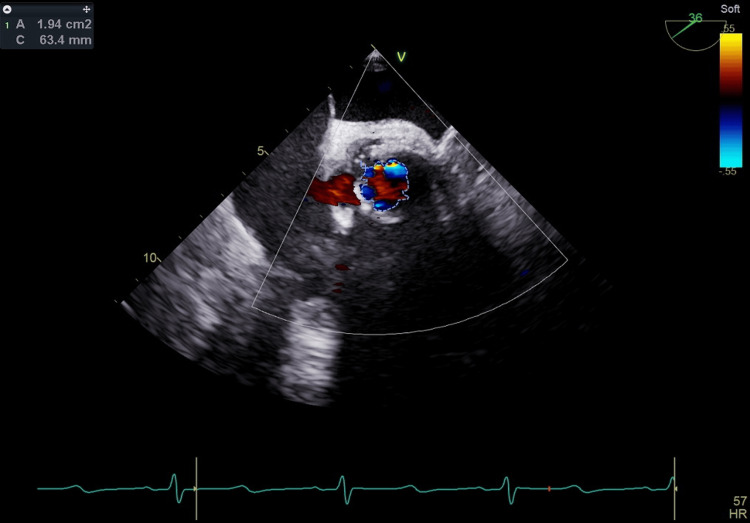
Assessment of the effective orifice area calculated by tracing the regurgitant orifice area on the aortic valve short-axis view of TEE after CPB It revealed an effective orifice area of 1.94 cm² by tracing the regurgitant orifice area on the aortic valve short-axis view of TEE. TEE: transesophageal echocardiography; CPB: cardiopulmonary bypass

After induction of anesthesia, we used TEE to confirm that cardiac function and the degree of AR remained largely unchanged from the findings of the preoperative TTE (Figure 1). Without administration of catecholamines, cardiac output (CO) was 4.3 L/min, cardiac index (CI) was 2.7 L/min/m², systemic vascular resistance (SVR) was 1191 dynes·s/cm⁵, the systemic vascular resistance index (SVRI) was 1896 dynes·s/cm⁵/m², and the hemoglobin level was 13.3 g/dL (Table 1). At that time, the BP was 103/43 mmHg by invasive blood pressure measurement, and HR was 75 beats per minute (sinus rhythm). After successfully completing the AVR and CABG, there was no LV wall motion. Stable hemodynamics were achieved, and cardiopulmonary bypass (CPB) was terminated once residual air in the left ventricle had resolved. Approximately 45 minutes after weaning from CPB, evaluation of the bioprosthetic valve on a mid-esophageal long-axis view on TEE revealed a maximum aortic valve blood flow velocity of 3.4 m/s, a maximum aortic valve pressure gradient of 45 mmHg, and a mean aortic valve pressure gradient of 23 mmHg, indicating findings corresponding to moderate aortic stenosis (AS) (Video 1; Figure 2). Under administration of norepinephrine 0.6 μg/kg/min and dobutamine 0.3 μg/kg/min, CO was 2.5 L/min, CI was 1.6 L/min/m², SVR was 2144 dynes·s/cm², SVRI was 3350 dynes·s/cm²/m², and hemoglobin was 9.7 g/dL (Table 1). At that time, the BP was 107/63 mmHg by invasive blood pressure measurement, and HR was 57 beats per minute (sinus rhythm). In addition to the artificial valve being in the normal aortic valve position, the angle formed with the mitral valve was 122 degrees. The effective orifice area index (EOAI), calculated by tracing the regurgitant orifice area on the aortic valve short-axis view, was 1.24 cm²/m² (Figure 3). The acceleration time (AT) of aortic valve blood flow was 42 ms, and AT/ejection time (ET) was 0.12. Since there was no aortic regurgitation or PPM, and TEE revealed enhanced left ventricular contractility compared to preoperative findings, we concluded that the AS-like findings were due to high left ventricular output. After consulting with the surgeon, we decided to monitor the patient.

**Table 1:** Cardiac output (CO), cardiac index (CI), systemic vascular resistance (SVR), and systemic vascular resistance index (SVRI) measured by FloTrac, as well as hemoglobin levels obtained from arterial blood gas analysis, at the time of induction of anesthesia and after weaning from cardiopulmonary bypass (CPB0. FloTrac, Edwards Lifesciences, Irvine, California, US

Measurement and test items	Reference range	After induction of anesthesia	45 minutes after weaning from CPB
CO	4.0-8.0 L/min	4.3	2.5
CI	2.5-4.0 L/min/m²	2.7	1.6
SVR	800-1,200 dynes·s/cm⁵	1191	2144
SVRI	1900-2400 dynes·s/cm⁵/m²	1896	3350
Hemoglobin	12.1-14.5 g/dL	13.3	9.7

**Video 1:** Accelerated blood flow corresponding to moderate AS assessed by the aortic valve long-axis view of TEE after CPB Accelerated blood flow corresponding to moderate AS was observed by TEE, and it revealed enhanced left ventricular contractility compared to preoperative findings. AS: aortic stenosis; TEE: transesophageal echocardiography; CPB: cardiopulmonary bypass

On postoperative day 0, the patient’s level of consciousness and respiratory status were not a problem, and since their hemodynamics were stable, without the need for catecholamine administration, the patient was extubated. A follow-up TTE on postoperative day 4 revealed an LVEF of 38 % and circumferential left ventricular wall motion impairment; however, aortic valve mobility was normal, and no regurgitation or stenosis was observed. The patient was discharged on postoperative day 19, without any neurological sequelae.

## Discussion

Although the development of moderate AS-like findings immediately following biological valve replacement is rare, it is a condition that poses significant challenges for clinical judgment and management [3].

In this case, moderate stenosis was observed immediately following AVR with a bioprosthetic valve. However, no surgical factors, such as displacement of the aortic valve seat or excessive suture contraction at the base of the ascending aorta, were identified [4]. Furthermore, since the EOAI was 1.24 cm²/m², which is greater than 0.85 cm²/m², the aortic valve provided an adequate effective orifice area relative to the patient’s body surface area. Given the stable hemodynamics and good leaflet mobility, it was determined that immediate re-replacement was not necessary [5]. Since the transit flow through the bioprosthetic valve of AT was 42 ms below 100 ms, valve stenosis was ruled out, and we thought the condition was attributed to high left ventricular output [6]. We believed this was influenced by a marked decrease in total peripheral vascular resistance due to the effects of CPB, left ventricular hypercontractility, and anemia caused by catecholamine administration [7,8]. Since FloTrac (Edwards Lifesciences, Irvine, California, US) estimates stroke volume based on the amplitude and rise time of the arterial pressure waveform, it is known that CO and CI are underestimated when peripheral vascular resistance decreases significantly due to the effects of CPB or sepsis, resulting in a flatter waveform [9,10]. Therefore, although a decrease in CO and CI was observed in this case following bioprosthetic valve replacement, we concluded that it was due to left ventricular hypercontractility based on TEE findings. Although this led to a contradictory conclusion, that “contrary to the FloTrac results, this is a case of left ventricular hypercontractility”, TEE findings proved useful for diagnosis, given that CO and CI values were unreliable at the time of CPB weaning.

In this case, because the long-axis view of the aortic valve through the stomach provided poor visualization, we evaluated the blood flow velocity of the prosthetic valve using a long-axis view of the aortic valve through the middle esophagus; however, due to the large angle correction, there is a possibility that this resulted in a significant discrepancy from the actual flow velocity [11]. Furthermore, while the diagnosis of left ventricular hypercontractility was initially based on AT and AT/ET values obtained from measurements of aortic valve flow velocity immediately following bioprosthetic valve replacement, along with TEE findings, the measurement of Doppler velocity index allows for an accurate assessment of whether left ventricular outflow tract flow velocity is being measured correctly, leading to a more precise differential diagnosis [12]. Furthermore, the finding of left ventricular hypercontractility was based on a visual assessment at the papillary muscle level using a mid-esophageal short-axis view of the left ventricle, which lacks the accuracy of quantitative evaluation.

## Conclusions

We reported a case in which moderate stenosis was observed in a bioprosthetic valve immediately following aortic valve replacement. Transesophageal echocardiography was useful in guiding the treatment plan for moderate aortic stenosis-like findings associated with high left ventricular output immediately following bioprosthetic valve replacement.
